# An Unroofing Method to Observe the Cytoskeleton Directly at Molecular Resolution Using Atomic Force Microscopy

**DOI:** 10.1038/srep27472

**Published:** 2016-06-07

**Authors:** Eiji Usukura, Akihiro Narita, Akira Yagi, Shuichi Ito, Jiro Usukura

**Affiliations:** 1 Structural Biology Research Centre, Graduate School of Science, Nagoya University, Nagoya, 464-8603 Japan; 3 Olympus Corporation, Hachioji, Tokyo, 192-8512 Japan

## Abstract

An improved unroofing method enabled the cantilever of an atomic force microscope (AFM) to reach directly into a cell to visualize the intracellular cytoskeletal actin filaments, microtubules, clathrin coats, and caveolae in phosphate-buffered saline (PBS) at a higher resolution than conventional electron microscopy. All of the actin filaments clearly exhibited a short periodicity of approximately 5–6 nm, which was derived from globular actins linked to each other to form filaments, as well as a long helical periodicity. The polarity of the actin filaments appeared to be determined by the shape of the periodic striations. Microtubules were identified based on their thickness. Clathrin coats and caveolae were observed on the cytoplasmic surface of cell membranes. The area containing clathrin molecules and their terminal domains was directly visualized. Characteristic ridge structures located at the surface of the caveolae were observed at high resolution, similar to those observed with electron microscopy (EM). Overall, unroofing allowed intracellular AFM imaging in a liquid environment with a level of quality equivalent or superior to that of EM. Thus, AFMs are anticipated to provide cutting-edge findings in cell biology and histology.

Binnig *et al.*^1^ first outlined the principle of the atomic force microscope (AFM) as a type of scanning probe microscopy. The AFM imaging method is fundamentally different from that of other microscopes. A sharp needle attached to a cantilever (the AFM probe) scans over the surface of a sample, and the topological structure is displayed based on measurements of the surface undulation. Subsequent technical developments by many investigators have revealed a wide range of biological applications for the AFM (see reviews^2^,^3^). Thus, the AFM has become a powerful tool to detect the surface structure of biological samples in liquid environments at a theoretical resolution comparable to that of electron microscopy (EM).

Unfortunately, AFM cantilevers cannot stably follow sudden changes in vertical undulations. Furthermore, several scanning instabilities originating from the surrounding liquid environment have limited the general use of atomic force microscopy in cell biology. In addition, an AFM has not been able to image intracellular structures because the cantilever cannot scan the inside of the cell membrane. Therefore, *in vivo* structural analysis using an AFM has been restricted to the outer surface of cell membranes. Indeed, even in their present state, AFM measurements in cells have not progressed very much, despite remarkable improvements in scanning speed and stability. The use of a high-resolution AFM has allowed the structural analysis of isolated and purified proteins and their complexes *in vitro*^4^,^5^,^6^,^7^,^8^,^9^, but not the *in vivo* or *in situ* analysis of intracellular structures. Most related atomic force microscopy studies have only detected the structure of the outer surface of cell membranes in a living or fixed state.

Recently, an unroofing technique has provided a way to view intracellular structures with an AFM^10^. Unroofing refers to breaking the cell membrane. When the cell membrane is broken, the intracellular region is exposed by removing the cytoplasmic-soluble components, which allows the AFM cantilever to access intracellular structures such as the cytoskeleton and organelles. This preparation technique, which uses sonication, was originally developed to observe membrane undercoats in freeze-etching EM^11^,^12^. Although rupture of the apical cell membrane is caused by the collision of micro air bubbles (cavitation) induced by sonication, the specimen manipulation for this technique requires meticulous care to prevent the detachment of whole cells from the coverslips. The current study presents a new unroofing apparatus consisting of an improved sonicator and light stereomicroscope that improves reproducibility and facilitates sample handling. The improved (custom-made) sonicator is able to generate cavitation at a much lower power (0.3 − 1 W) than that of the commonly used sonicator (50 W or higher). We used this method in an atomic force microscopy analysis of the intracellular cytoskeleton in phosphate-buffered saline (PBS) at a molecular resolution. Furthermore, to improve the signal-to-noise (S/N) ratio and thereby refine the images obtained with the AFM, serial scanned images of a target organelle were averaged using mathematical calculations.

## Results

**Figure 1:**
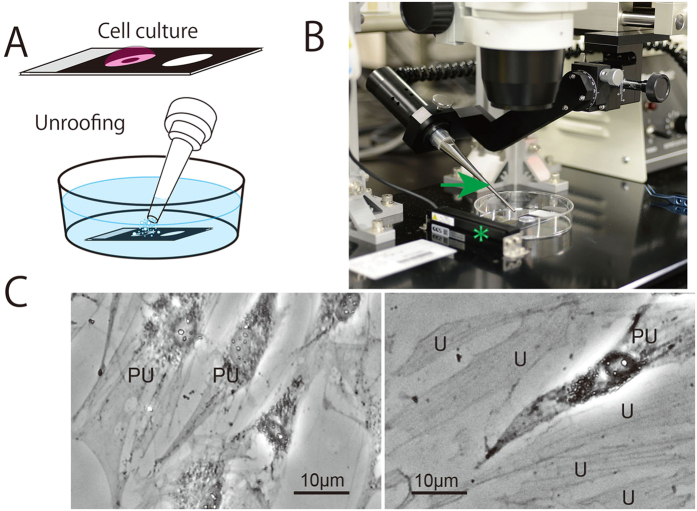
(**A**) A glass slide modified with a hydrophobic print was used in all of the procedures, from the cell culture to the AFM measurements. The samples were unroofed by micro bubbles generated from the tip of a sonication probe when the glass slide was placed under the buffer solution. (**B**) The improved unroofing apparatus consists of a low-power sonicator (custom-made) and stereomicroscope equipped with a position controller for the sonication probe and an LED light-sheet illumination system (asterisk) to observe the unroofing process. An arrow indicates probe of sonicator. (**C**) Image of unroofed NRK cells using a phase-contrast microscope. PU indicates a partially unroofed cell in which the organelles and nucleus are retained. U indicates entirely unroofed cells in which the organelles and nuclei have been removed but the cytoskeletons are retained.

Unroofing is a very useful method to expose the inside of cells and thereby enable intracellular observations via various microscopic techniques, but there are currently few methods that are easy to use with good reproducibility. Although chemical unroofing methods using detergents such as Triton X 100 are easy to use, interactions between the cytoskeleton and membrane are never observed because the membrane and organelles are completely dissolved. A membrane-breaking method using changes in osmotic pressure was inefficient and did not work in some cells in our experience. In an earlier study^13^, the authors were not able to visualize the cytoskeleton and organelles inside the cell at high resolution. In contrast, the unroofing method using sonication is particularly good for freeze-etching EM^11^,^12^. However, the reproducibility is not good because whole cells can frequently be detached from the glass surface during sonication under the original conditions (10 to 50 W). Even if the cell membrane remains on the glass after unroofing, many of the cytoskeletal components and organelles are completely removed. Therefore, we developed a reliable unroofing device. First, an ultrasonic generator was improved to precisely regulate the output power in the range of 0 − 1 W. This custom-made low-power sonicator was further combined with a stereomicroscope equipped with a position controller for the sonication probe and an LED light-sheet illumination system to observe the unroofing procedure in detail (Fig. 1B). After these modifications, this apparatus enabled us to unroof cells to varying degrees, from the partial to entire removal of the cytoplasm (Fig. 1C).

**Figure 2:**
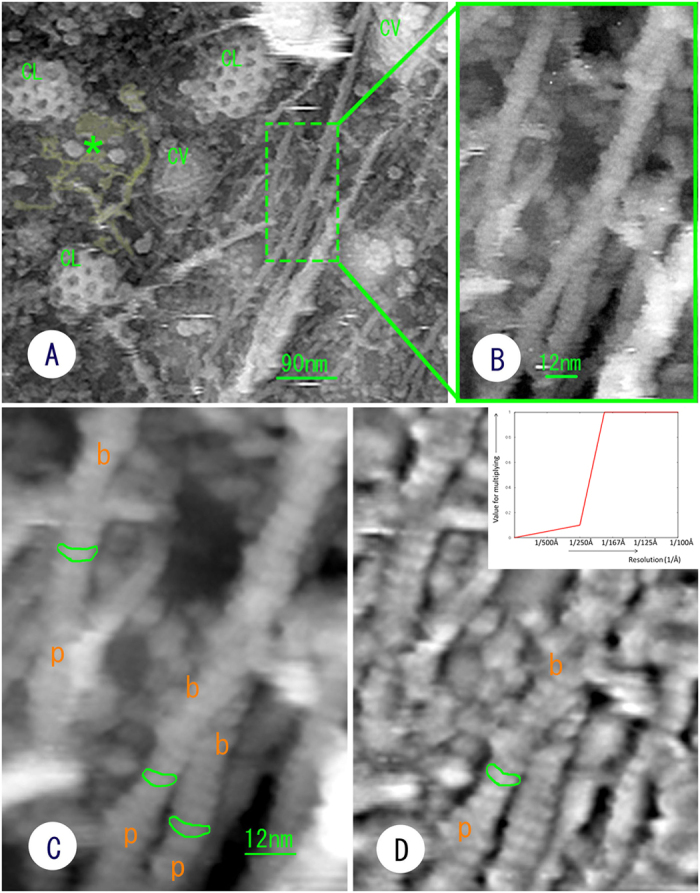
(**A**) AFM image of the cytoplasmic surface of the cell membrane exposed by unroofing at a suitable power. Actin filaments extend along the inside surface of the cell membrane. A clathrin coat (CL), caveolae (CV) and an ER-like membranous structure (asterisk and painted with pale yellow) are observed. (**B**) High-magnification image of the boxed area in image (**A**). All the actin filaments have a short periodic striation. (**C**) Averaged image of 10 consecutive raw scanning images of the same area as in image (**B**). The periodic elements appear to be slightly curved (highlighted with green lines). (**D**) Enhanced image of image (**C**), which was created through a filter to enhance the high-resolution signal (a type of high band-pass filter: inset). p: pointed end. b: barbed end.

**Figure 3:**
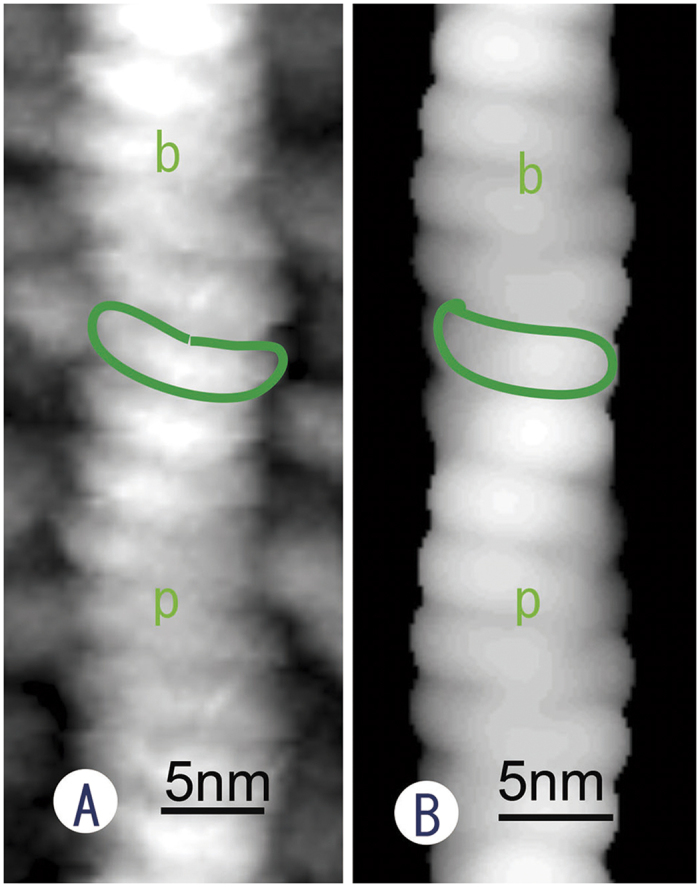
(**A**) AFM image of an actin filament polymerized *in vitro* from purified G-actin. Short, periodic, slightly curved striations were observed much more clearly (highlighted with a green line) than in the *in vivo* observations. Polarity was estimated based on the curved face of the periodic striations. (**B**) Image simulated from an atomic model of an actin filament. The polarity of the atomic model of an actin filament reflects the simulated image as a curved striation (indicated by a green line), revealing the polarity of the filament. The concave face of the striation has a barbed end. The polarity of the filaments is determined from the direction of the curvature. p: pointed end, b: barbed end.

The cantilever was thus able to directly reach the cell interior. Unlike conventional AFM measurements from outside the cell membrane, single actin filaments, bundles of actin filaments (stress fibres), clathrin coats, caveolae and so on were observed at the cytoplasmic surface of the cell membrane (Fig. 2A), yielding images that were similar to the those obtained via freeze-etching EM. Upon increasing the magnification, each actin filament in the stress fibre was discernible, revealing a short periodicity derived from globular actin (G-actin) that assembled to form filaments (Fig. 2B). This periodicity varied from 4.5 to 6 nm along the long axis of a single actin filament, with an average interval of 5.4 nm, as obtained by Fourier transformation. As depicted in Fig. 2A, it should be noted that these structures are displayed at an obviously high resolution that is comparable to EM, although the measurements were performed in a liquid environment (PBS). With careful observation, the periodic striation appeared to be slightly curved because the two G-actin molecules that formed each striation were oriented at different angles. The striation became more evident upon averaging the images (Fig. 2C,D). Based on the direction of the curve, the polarity of the actin filaments could be determined. To assess whether the slightly curved shape of the short periodic striations in the actin filaments is a real structure and whether the curved form virtually reflects the molecular structure of the actin filament, we first observed actin filaments that were polymerized from purified G-actin^14^ and then simulated how the actin filaments should be displayed by an AFM^15^,^16^. The *in vitro* polymerized actin filaments had the same structure as those observed *in situ* (Fig. 3A). This result confirms that the AFM measurements in this study provided accurate structural information about the actin filaments. In addition, the simulated image was quite similar to that obtained experimentally, as shown in Fig. 3B. Namely, it is evident that the curved shape of the periodic striation reflects the molecular structure of the actin filaments. When comparing the simulated image with the molecular model, the concave side of the curved striation appears to correspond to the barbed end. Therefore, the polarity of the actin filament could be determined *in situ* when the shape of the striation appeared curvilinear at high magnification. We tentatively marked the polarity in Fig. 2C,D according to this judgment. Further experiments may be required to confirm the polarity in a more concrete way. However, one of the co-authors, Narita, and colleagues^17^ succeeded in determining the polarity of actin filaments in a similar manner using EM.

In contrast, the thickness of the actin filaments detected with the AFM was variable and ranged from 5 nm to 15 nm. This may be partly because the thickness of the filaments is modified by the diameter of the tip of the scanning probe and reduced by the position of the probe relative to the sample. Therefore, an AFM may be not as reliable in measuring actin filament thickness.

**Figure 4:**
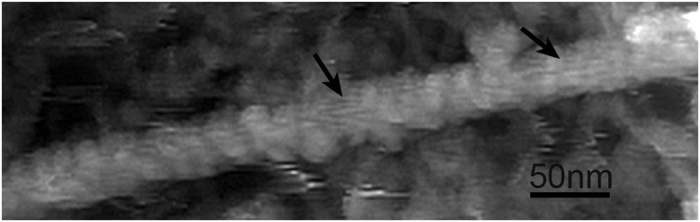
AFM image of a microtubule, that was judged by the total shape and thickness. However, constituent proto-filaments were not observed clearly, because microtubules were covered with many associated-proteins. A mechanical scratch by repeated scanning with the AFM tip exposed several striations (arrows), but it is not obvious whether they are proto-filaments.

Microtubules with a diameter of approximately 24 nm were observed to accompany actin filaments or stress fibres in the cytoplasm (Fig. 4). Unfortunately, proto-filaments were not easily detected *in situ*. This is partly because the microtubules in the cytoplasm are modified with several microtubule-associated proteins (Fig. 4). When the surface of a microtubule was scratched mechanically with the scanning tip, longitudinal striations resembling proto-filaments became visible (Fig. 4). However, the actual structure of microtubules in a liquid environment requires future study.

**Figure 5:**
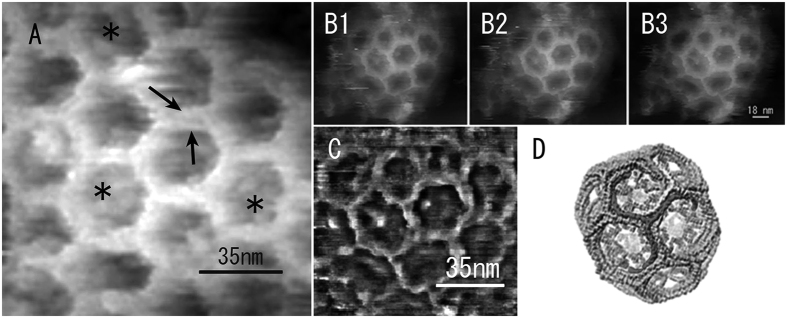
(**A**) High-magnification, raw AFM image of the clathrin coats recorded in PBS. Part of the triskelion shape of the clathrin molecules can be traced (indicated by arrows). The terminal regions of the clathrin molecules are partially visible in the centre of the hexagonal mesh (asterisks, see text for details). B1, B2, and B3: Consecutive, raw, scanned images of the same clathrin coat. (**C**) Averaged image of those in B1–B3, with high-resolution enhancement. The averaged image resembles the clathrin coat model proposed by Fotin *et al.*^18^ (**D**).

**Figure 6:**
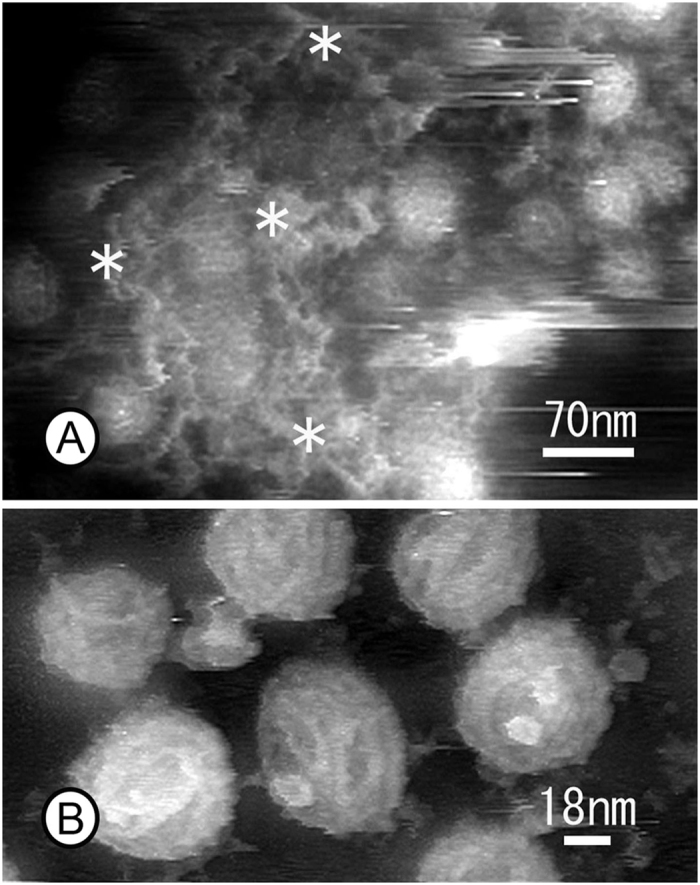
(**A**) AFM images of clusters of caveolae covered with a tubular meshwork of smooth ER-like structure (asterisks) on the inside surface of membranes. (**B**) High-magnification image of caveolae in which the apical surfaces are covered with a striated coat.

Clathrin coats and caveolae were frequently detected on the cytoplasmic surface of cell membranes, as revealed in Fig. 2A. Clathrin coats were relatively stable against repeated probe scanning in PBS due to the characteristic hexagonal lattice structure of these features. Therefore, the portion of the clathrin coats that contained the triskelion shapes of the clathrin molecules and their terminal domains could be observed at higher magnification (Fig. 5A). The visible region that could be traced seems to be the proximal segment of the heavy chain based on the molecular model produced by Fotin *et al.*^18^. Because the terminal regions are located in the centre of the hexagonal meshwork in this model, some blobs observed in the centre of the hexagonal mesh are thought to be the terminal regions of the clathrin molecules. The quality of the AFM images seems to be better than images of freeze-etched clathrin coats that have been taken to date using EM. We also averaged three consecutively scanned images (Fig. 5B1, 2, 3) to enhance the details of the clathrin coats, as described in Fig. 5C. The cytoplasmic surface of the cell membrane also contained caveolae and sometimes clusters of caveolae (Fig. 6A), which are easily identified by their characteristic ridge structures on the surface^19^,^20^ (see supplemental Fig. S1). Clearly, the ridge structures on the surface of the caveolae visualized with the AFM at high magnification (Fig. 6B) are similar or superior to those visualized using freeze-etching EM, even though the images were obtained in a liquid environment.

As mentioned above, conventional sonication (10 W or more) results in the loss of many whole cells. Even if cells are unroofed successfully, neither the organelles nor nuclei would be attached. Only a few cytoskeletons are attached on the ventral cell membrane that remains on the glass. Although the ablation of the cytoplasm by unroofing is not constant even at the same sonication power, sonication affects not only the amount of remaining cytoskeleton but also appears to change the types of remaining organelles. Indeed, mild unroofing revealed the existence of membranous structure just beneath the cell membrane (Figs 2A and 6A). It should be noted that this membrane system was located over the caveolar region where tubular networks are formed (Fig. 6A). This is very interesting when considering the function of the membrane structure. However, the precise identification of this structure remains for future work.

## Discussion

**Figure 7:**
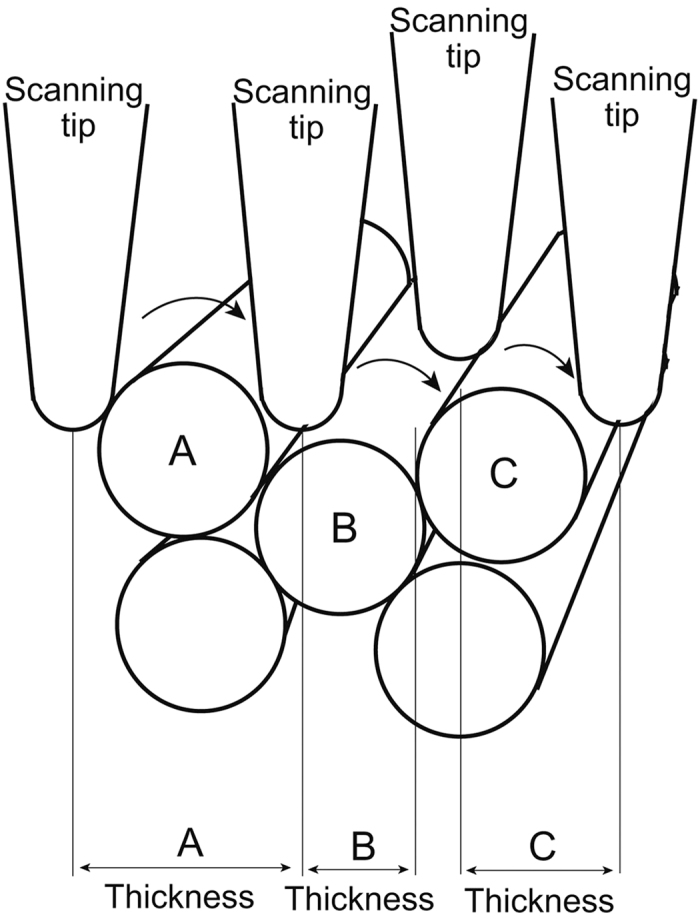
Illustration showing AFM measurement of the bundle of fibre. The thickness of each filament depends on the sharpness of scanning tip and the spatial position relative to the filament. Even if (**A**–**C**) are the same kind of filaments with the same thickness, thickness measured by AFM is different each other depending on the spatial position of scanning tip over the sample.

In cell biology, *in vivo* AFM imaging has long been limited to surface observations of living or fixed cells at a slightly higher resolution than that of light microscopes. There has been a large gap between the theoretical resolution and biological applications of AFM, partly because there have been no appropriate sample preparation methods for atomic force microscopy. Many studies have investigated cortical actin filaments with an AFM, but unfortunately, these studies involved indirect observations through cell membranes^21^,^22^,^23^,^24^,^25^,^26^,^27^,^28^,^29^,^30^,^31^,^32^,^33^,^34^. In these studies, bundles of actin filaments (stress fibres) were only observed as several linear disturbances or ridges on the surface of cells. Therefore, stress fibres were suggested to be producing the images based on comparisons with fluorescence microscopy data and/or the measurement of the width of the linear disturbances. Considering that the cortical cytoskeleton consists of actin filaments, microtubules and intermediate filaments, it is impossible to identify individual actin filaments through the cell membrane. In this regard, several studies used a detergent such as Triton X to remove the cell membrane and successfully observed intracellular structures^35^. However, the detergent completely dissolved the membrane system, including the organelles; therefore, the membrane undercoat, clathrin coats, caveolae, and the interactions between the cytoskeleton and cell membrane were never observed. The unroofing method makes it possible for the AFM cantilever to directly access intracellular organelles, while maintaining the ventral cell membrane in its native state. Indeed, actin filaments, clathrin coats and other structures could be detected at the expected resolution. In practice, the short periodicity of the actin filaments along the long axis was observed more clearly with the AFM than by freeze-etching EM because the sharp tip of the cantilever traces the surfaces of the actin filaments while in contact with these structures during atomic force microscopy. In contrast, the surfaces of actin filaments are evaporated with platinum and carbon in a freeze-etching replica in EM. This is why an AFM can clearly describe the short periodicity of actin filaments. However, determination of the thickness of the filaments may be not reliable because the tip diameter and the position of the cantilever needle in the z axis changes the apparent thickness of the filaments (see the illustration in Fig. 7). With a freeze-etching replica in EM, optimal shadowing is necessary to visualize the periodicity of actin filaments. Even if the periodicity of the actin filaments became apparent, their polarity could not be detected without myosin S1 decoration because metal shadowing covered the slight curvature of the periodic striation. This is also true for the triskelion shape of the clathrin molecules. Clearly, the ability of the AFM to observe this fine structure is an advantage over EM.

Atomic force microscopy has not been able to depict intracellular structures at theoretical resolution until the removal of the apical cell membrane by unroofing techniques. In practice, *in situ* images at the same resolution as those in this study have not previously been taken with any type of microscope. Because the current images were obtained in PBS, the structures that contain water molecules can be imaged. Therefore, the current results are valuable for understanding the actual structure of cytoskeletons and organelles in cells, revealing the utility of the developed unroofing technique for future studies in cell biology.

However, it is not simple to regulate the degree of unroofing to visualize the surface of intracellular organelles. The unroofing technique was originally developed to observe the cytoplasmic surface of membranes (the membrane undercoat) in freeze-etching replica EM^10^,^11^. Therefore, relatively strong sonication might be acceptable in certain cases because the complete removal of soluble proteins was necessary to reveal membrane-attached structures such as clathrin coats. Commercial sonicators are typically manufactured for homogenization or cleaning; therefore, their output power is usually approximately 50 W or higher at a frequency of 27 KHz. Under such conditions, whole cells are detached from the glass surfaces and fragmented. Therefore, it is necessary to decrease the power considerably to promote effective unroofing. For this purpose, we developed with the assistance of Tomy Seiko (Tokyo, Japan) a sonication device that is capable of precisely regulating the sonication power from 0 to 1 W at a frequency of 27 KHz (custom-made, Patent pending). During our study, it became clear that membrane rupture is induced by micro bubbles (cavitation), particularly at lower power. The tip of the sonication probe was scratched to form a shallow groove on the surface to produce sufficient cavitation.

A reduction of the output power also seemed to decrease the artefacts of unroofing, including breaking the organelle membranes, removing too much cytoskeleton and fragmentation. Moreover, the unroofing artefacts produced by low-power sonication appear to be less than those produced by chemical unroofing, such as Triton X treatment or biochemical isolation procedures. Low-power sonication is similar to sectioning, although the soluble substances are washed away upon the disruption of the cell membrane. This type of unroofing could be used as a preparation method in freeze-etching EM, cryo-EM, SEM and light microscopic immunocytochemistry. Whereas unroofing has been successfully used for these microscopy techniques, we will continue to validate its use for AFM studies to avoid any potential artifacts. Low-power unroofing allowed us to image fragile membrane structures in this study, although the structures were still fragmented, as indicated in Fig. 2A (asterisk and area painted yellow) and Fig. 6A (asterisks). We assumed that the fragmented membrane structures possible represented smooth endoplasmic reticulum (smooth ER). Their precise identification requires future study. Although the smooth ER is well developed in muscle cells (Ca^2+^ regulation) and adrenal cells (steroid synthesis), the spatial localization of the smooth ER in non-specialized cells is not well known at present. In these tissues, the smooth ER is characterized by a very flat, thin and tubular meshwork. Sometimes, this membrane structure was observed just beneath the cell membrane in cryo-EM and freeze-etching EM (see supplemental Fig. S2).

Recently, mathematical averaging of images has become popular in single-particle analyses of purified proteins by negative staining or cryo-EM. In the case of *in vitro* imaging using purified proteins, there are no problems with averaging the images of different particles (proteins). However, many proteins, particularly filaments, are modified *in vivo* with several proteins to function properly at different locations in cells. Therefore, averaging the images of target proteins localized at different places within a cell results in the loss of minor structures or the actual structure. In this study, we applied an averaging method to consecutive, scanned images of the same filament or structure. This improved the S/N ratio of the images such that the short periodicity of the actin filaments was refined and useful to determine the polarity of the filaments. The images averaged from three consecutively scanned images of clathrin coats were also well refined compared with the raw images. Therefore, averaging scanned images seems to be effective for structural analysis.

Although freeze-etching EM is a unique method for describing three-dimensional cytoskeletons, the images reveal a surface topology similar to atomic force microscopy because of the necessary shadowing with platinum and carbon. The image resolution is limited by the amount of shadowing and does not equal the resolution of EM. In contrast, the scanning tip of an AFM is able to trace the surface undulation laterally at the theoretical resolution, which is limited only by vertical instability. If the samples are prepared appropriately, for example via unroofing as shown in this study, an AFM is able to describe intracellular structures at molecular resolution. However, measured values, such as the filament thickness, depend on the relative position of the scanning tip to the sample in addition to the tip radius, as illustrated in Fig. 7. Filaments located in the uppermost layer appeared thicker than their true thickness, while filaments deeper in the sample appeared thinner because of partial tracing. However, individual actin filaments were discernible in stress fibres, as illustrated in Fig. 2B. Another major disadvantage of the AFM is that its findings are not applicable to those of immunocytochemistry. Therefore, we cannot identify the constituent substances in the structure. In freeze-etching EM, some actin filaments appeared to be fused firmly with each other in the stress fibre, which is attributed to shadowing and/or the differences in the observation environments: freeze-drying for EM and a liquid environment for AFM. Overall, the developed unroofing sample preparation approach allows different imaging techniques to provide complementary data on biological samples.

## Methods

### Cell culture

Glass slides containing hydrophilic circular windows with a diameter of 15 mm surrounded by a framework of printed hydrophobic black ink (TF0215; Matsunami Glass Industry, Ltd., Osaka, Japan) were used for both cell culture and the AFM measurements. When culture medium (D-MEM: Sigma-Aldrich Co. St. Louise, MO, USA) was added to this hydrophilic circular window, the medium produced droplet because of the hydrophobicity of the surrounding framework, as depicted in Fig. 1A. Normal rat kidney (NRK) cells were cultured in this swelled medium for 2 days in a CO_2_ incubator and then used in the subsequent experiments. To move the cantilever exactly onto the target area in the cells, a thin sapphire disc (3 mm in diameter, 0.05 mm thick; Rudolf Brügger, Swiss Micro Technology, Lugano, Switzerland) with coordinates was glued in the centre of each hydrophilic circle prior to cell culture. The coordinates on the sapphire discs were printed by carbon shadowing after an EM finder grid was placed on the disc.

### Unroofing

To observe the intracellular cytoskeleton and the other organelles, the following unroofing method that used sonication was performed. Cells cultured on glass slides were sequentially washed with Ringer’s solution consisting of 155 mM NaCl, 3 mM KCl, 2 mM CaCl_2_, 1 mM MgCl_2_, 3 mM NaH_2_PO_4_, and 10 mM glucose in 5 mM HEPES buffer (pH 7.4) followed by Ca^2+^-free Ringer’s solution (lacking CaCl_2_). The cells were soaked for approximately 10 s in a polylysine solution (Mw 30,000–50,000; Sigma-Aldrich, St. Louis, MO, USA; 0.5 mg/ml dissolved in Ca^2+^-free Ringer’s solution) and then washed three times for a few seconds each in hypotonic Ringer’s solution, which was prepared by mixing one part Ca^2+^-free Ringer’s solution with two parts distilled water. This step swelled the cells, enabling them to burst easily during sonication. Immediately after washing with the hypotonic solution, the samples were placed approximately 2 mm from the tip of the sonicator probe and exposed for 5–10 s to fine air bubbles (cavitation) generated by weak sonication (0.5 W, 27 KHz) in isotonic buffer^11^,^12^ consisting of 30 mM HEPES, pH 7.4, 70 mM KCl, 3 mM MgCl_2_, 1 mM EGTA, 1 mM DTT and 0.1 mM 4-(2-aminoethyl) benzene sulfonyl fluoride hydrochloride (AEBSF, Pefabloc SC: Roche Diagnostics Gmbh, Mannheim Germany) as a protease inhibitor. The unroofed cells were washed briefly in the same fresh buffer and immediately fixed with 2% glutaraldehyde in the same buffer for 20 min. The fixed samples were washed twice with PBS and then examined with the AFM. The ultrasonic generator (Tomy Seiko Co., Tokyo, Japan) was custom-made to regulate the power in the range between 0 to 5 W and assembled with a stereo-light-microscope that was specifically designed (Olympus Co., Tokyo, Japan) to observe the unroofing process with light-sheet illumination. Light-sheet or low-angle illumination (nearly 0°) is important to clearly view both the micro bubbles generated by sonication and the cells attached to the glass slide. The final instrument is described as an unroofing apparatus and is depicted in Fig. 1B (Patent pending). In this study, the sonication power was 0.3 to 0.5 W.

This apparatus enabled us to observe various intracellular organelles by changing the degree of unroofing. The condition of the unroofed cells was checked with a phase-contrast light microscope. Partially and/or well-unroofed cells are presented in Fig. 1C.

### AFM measurements

The glass slide containing unroofed cells soaked in PBS was assembled in a tip-scan high-speed AFM imaging system that was improved based on a previously developed AFM^38^. This AFM unit was combined with an inverted fluorescence microscope (IX73 Olympus Corporation, Tokyo) and named BIXAM. Therefore, the probe cantilever was depressed slowly onto the target region of the cells while observing the cells under a light microscope. The AFM was used in the phase-modulation mode for high-resolution imaging^39^. A characteristic of this AFM is to adopt small and soft cantilevers (2 μm wide, 9 μm long, and 0.1 μm thick in size) with a spring constant of 0.1 N/m (BL-AC10DS, BL-AC10EGS, Olympus Corporation). The resonance frequency of the probe tip is 1.2 MHz in air and approximately 400 kHz in water. The diameter of the scanning tip was approximately 8–10 nm. This AFM (BIXAM) acquires a sample image with a characteristic cantilever in the phase modulation mode in an amplitude domain of approximately 1–5 nm. When image measurements were performed in the phase modulation mode, it is unreasonable to estimate the set force. However, provided that the probe amplitude is approximately 5 nm in water, and it is decreased by approximately 10% upon the interaction between the probe and sample, the force against samples is estimated to be 50 pN. The maximum scanning range of the device was 4 μm (x axis) × 3 μm (y axis), but the range of 200–600 nm was primarily scanned in the present study. The scanning resolution was 320 pixels (x axis) × 240 lines (y axis).

### Image analysis

To improve the S/N ratio of some images, computer-assisted averaging was examined for the first time in atomic force microscopy. More than three sequential frames taken at the region of interest were averaged, while considering drifting and rotation. EOS software was used for the averaging^15^. To obtain detailed information from the AFM images by enhancing the high-resolution signal by more than 18 nm, some images were multiplied by a filter in Fourier space (Fig. 2D Inset).

### AFM observation of purified actin

Actin was purified from rabbit skeletal muscle as previously described^14^. Actin (0.1 mg/ml) was incubated for 30 min at room temperature in a solution containing 50 mM NaCl, 10 mM sodium phosphate buffer (pH 7.4), 1 mM MgCl_2_ and 1 mM DTT. To increase its affinity for the actin filament, a glass slide (TF0215; Matsunami Glass Ind., Ltd., Osaka, Japan) was coated with polylysine as described below. The glass slide was washed twice with ethanol, a polylysine solution (Mw 30,000–50,000; Sigma-Aldrich, St. Louis, MO, USA; 0.4 mg/ml dissolved in water) was applied, and then the slide was washed three times with the buffer. Polymerized actin was diluted 10 times with buffer containing 1 μM phalloidin and applied to the polylysine-coated glass slide. The AFM measurement was performed as described above.

### Generating the simulated images

EOS software was used again for the image simulation^15^. The actin filament model^16^ was placed along the y axis in the computer. As a model for the AFM tip, a sphere with a diameter of 8 nm was generated at an (x, y, z) coordinate. The initial z value was larger than the maximum z position in the actin filament model by 8 nm. Then, z was decreased until the sphere touched the actin filament model. The final z was plotted as a pixel value at (x, y) in the simulated image. When 0.5% of the volume of the sphere overlapped with the actin filament model, we considered that the sphere touched the actin filament. The radius of the sphere was determined such that the simulated images resembled the observed images.

## Additional Information

**How to cite this article**: Usukura, E. *et al.* An Unroofing Method to Observe the Cytoskeleton Directly at Molecular Resolution Using Atomic Force Microscopy. *Sci. Rep.* **6**, 27472; doi: 10.1038/srep27472 (2016).

## Supplementary Material

Supplementary Information
