# Supplementary material for: An Unroofing Method to Observe the Cytoskeleton Directly at Molecular Resolution Using Atomic Force Microscopy
Source: Sci Rep. 2016 Jun 7;6:27472. doi: 10.1038/srep27472 (PMC4895337; doi:10.1038/srep27472)
Supplement: Supplementary Information [file srep27472-s1.doc]

Supplementary Information

*Manuscript title:*

An Unroofing Method to Observe the Cytoskeleton Directly at Molecular Resolution Using Atomic Force Microscopy.

*Authors list:*

Eiji Usukura^1,*^, Akihiro Narita^1,*^, Akira Yagi^2,*^, Shuichi Ito^2,*^ and Jiro Usukura^1,*^

1. Structural Biology Research Center, Graduate School of Science, Nagoya University, Nagoya, 464-8603 Japan
2. Olympus Corporation, Hachioji, Tokyo 192-8512 Japan

^*^These authors contributed equally to this work.

Corresponding author: usukuraj@esi.nagoya-u.ac.jp

Supplementary Figures

Supplementary figure S1


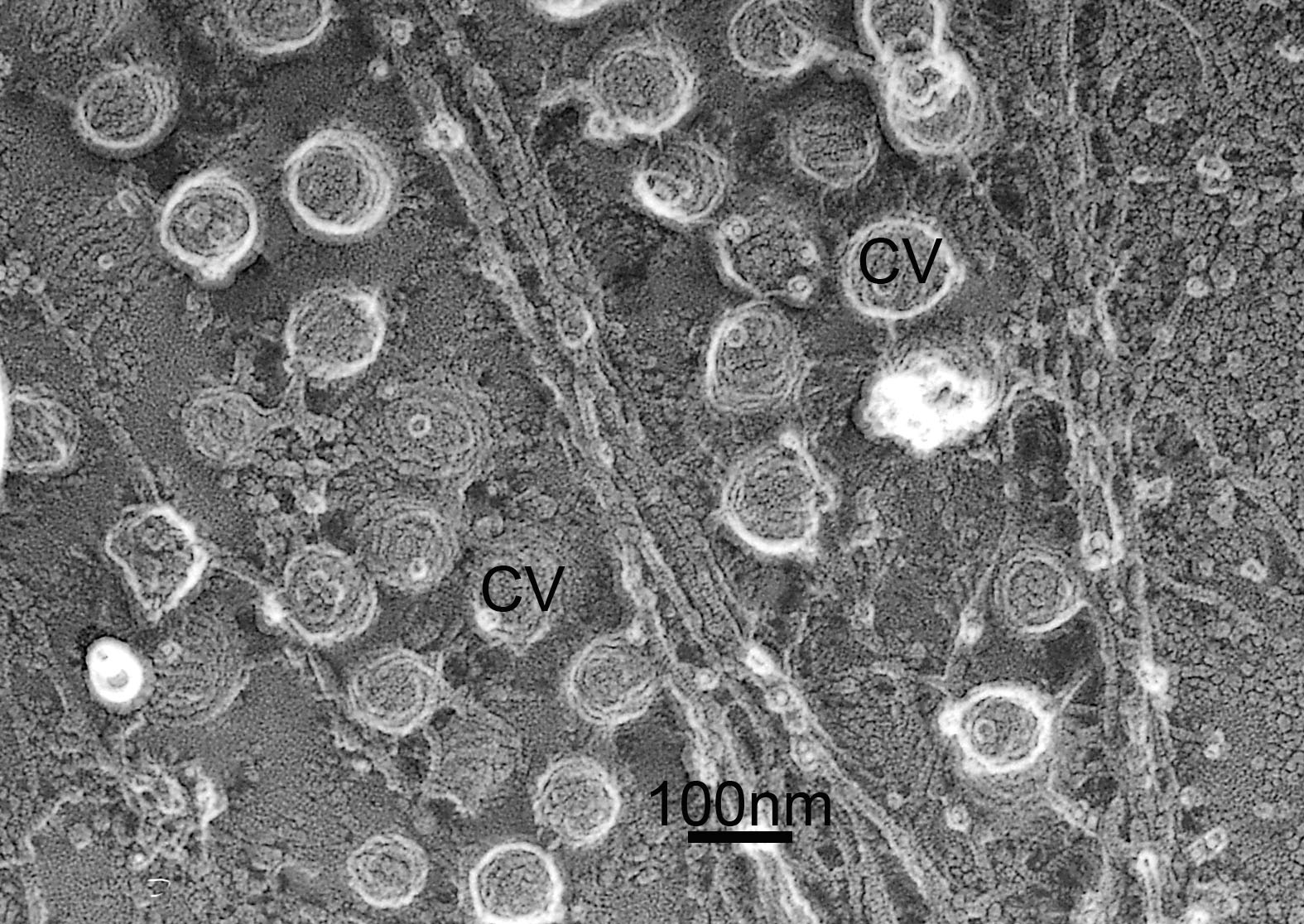


Fig. S1. Freeze-etched replica EM showing caveolae (CV), whose surface is modified with characteristic ridges.

Supplementary Figure S2


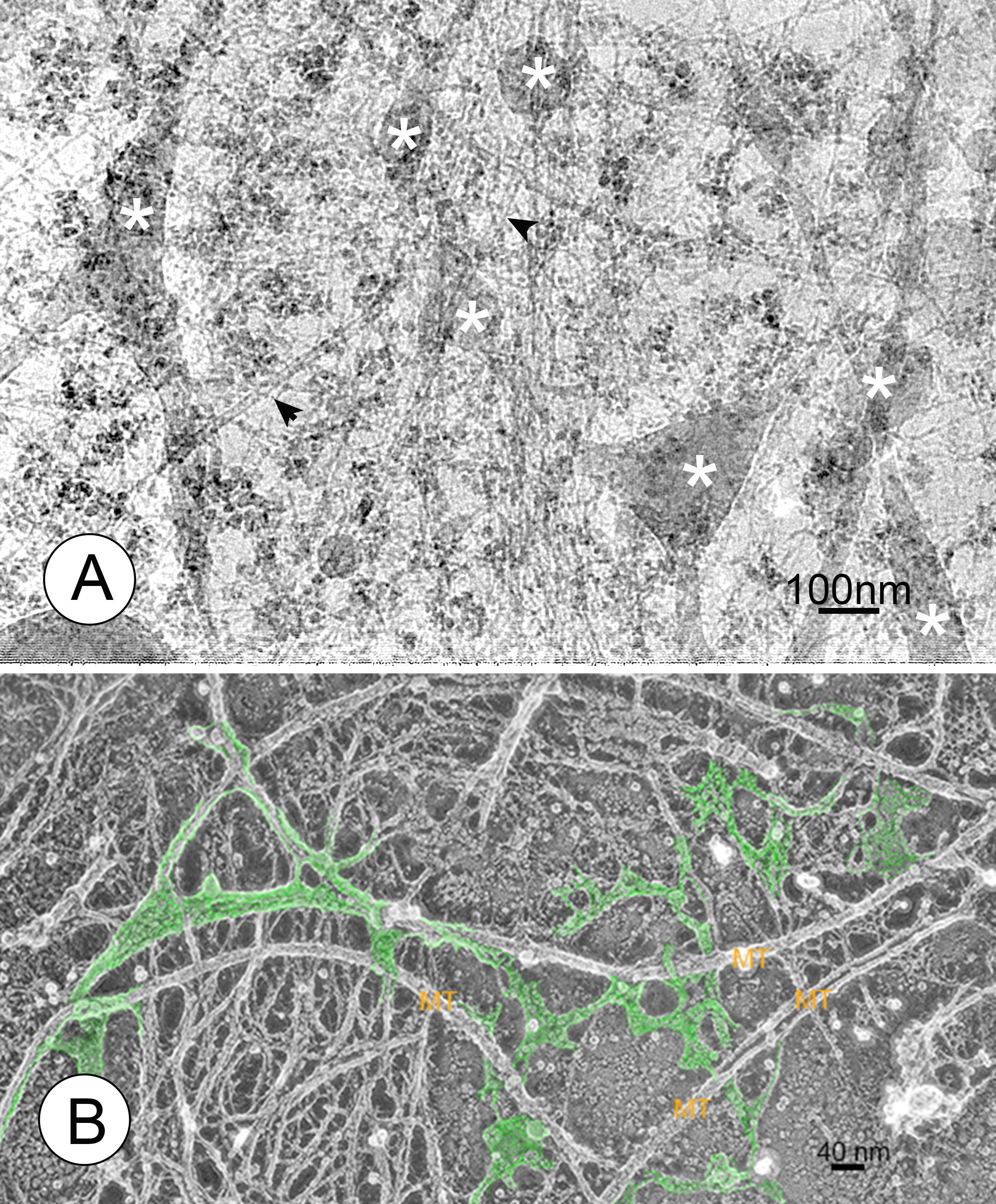


Fig S2. A: Cryo-electron micrograph of unroofed cell in native state showing cytoskeleton and membrane network (asterisks) while over rapping each other. Arrow heads show microtubules.

B: Freeze etching electron micrograph of membrane cytoskeleton. Membranous network consisting of tubular and flat sac (painted with green) is also recognized here while associating with cytoskeletons. MT: microtubule. These membranous structures possible represents smooth ER
